# Supplementary material for: Long-term safety and clinical outcomes from a single-site phase 1 study of neural stem cell transplantation for chronic cervical spinal cord injury
Source: Stem Cell Reports. 2026 May 21;21(6):102926. doi: 10.1016/j.stemcr.2026.102926 (PMC13261865; doi:10.1016/j.stemcr.2026.102926)
Supplement: Document S1. Table S1 [file mmc1.pdf]

Stem Cell Reports, Volume *21*

Supplemental Information

Long-term safety and clinical outcomes from a single-site phase 1 study of neural stem cell transplantation for chronic cervical spinal cord injury

Mickey E. Abraham, Joel R. Martin, Margaret Seaton, Michael G. Brandel, Kiefer J. Forseth, Catriona Jamieson, Martin Marsala, and Joseph D. Ciacci

Supplemental Table 1. ***Clinical outcomes of included patients.*** NLI=Neurological Level of Injury; FIM=Functional Independence Measure (out of 128); SCIM=Spinal Cord Independence Measure (out of 100); "-" indicates testing was not done

	Baseline	*4wk*	*12wk*	*6m*	*12m*	*18m*	*36m*	*42m*	*48m*	*54m*	*60m*
0202											
ISNCSCI											
R Sensory	C8	C8	C8	C8	C8	C8	C7	C7	C7	C7	C7
L Sensory	C8	C8	C8	C8	C8	C8	C7	C7	C7	C7	C7
R Motor	C7	C7	C7	C7	C7	C7	C7	C8	C8	C8	C8
L Motor	C7	C7	C7	C7	C7	C7	C7	C8	C8	C8	C8
NLI	C7	C7	C7	C7	C7	C7	C7	C7	C7	C7	C7
HLA	-	-	Neg	Neg	Neg	Neg	-	-	-	-	-
FIM	100	-	-	106	109	112	-	-	-	-	-
SCIM	58	-	-	58	64	64	64	64	55	56	63
Avg Pain	3	5	3	2	2	3	2	1	2	1	1
EMG		No Δ	No Δ	No Δ	No Δ	No Δ	No Δ	No Δ	No Δ	No Δ	No Δ
BMCA		No Δ	No Δ	No Δ	No Δ	No Δ	L wrist flex/ext	L wrist flex/ext	L wrist flex/ext	L wrist flex/ext	L wrist flex/ext
0203											
ISNCSCI											
R Sensory	C6	C6	C6	C6	C6	-	-	C6	-	C5	-
L Sensory	C6	C6	C6	C6	C6	-	-	C6	-	C6	-
R Motor	C7	C7	C6	C7	C7	-	-	C7	-	C6	-
L Motor	C6	C6	C6	C6	C6	-	-	C6	-	C5	-
NLI	C6	C6	C6	C6	C6	-	-	C6	-	C5	-
HLA	-	-	Neg	Neg	Neg	-	-	-	-	-	-
FIM	58	-	-	58	59	-	-	-	-	-	-
SCIM	22	-	-	21	20	-	20	26	23	29	-
Avg Pain	6	4	-	-	7	-	8	7	6	7	-
EMG		No Δ	No Δ	R deltoid MUAPs	R deltoid MUAPs	-	-	R deltoid MUAPs	-	R deltoid MUAPs	-
BMCA		No Δ	No Δ	No Δ	R biceps, triceps, wrist ext	-	-	R biceps, triceps, wrist ext	-	R biceps, triceps, wrist ext	-
0206											

ISNCSCI											
R Sensory	C6	C6	C6	C6	C6	-	-	C6	C5	C7	C7
L Sensory	C6	C6	C6	C6	C7	-	-	C6	C5	C6	C6
R Motor	C6	C6	C6	C6	C6	-	-	C6	C6	C6	C6
L Motor	C6	C6	C6	C6	C7	-	-	C6	C6	C6	C6
NLI	C6	C6	C6	C6	C6			C6	C6	C6	C6
HLA	-	-	Neg	Neg	Neg	-	-	-	-	-	-
FIM	75	-	-	83	86	-	-	-	-	-	-
SCIM	38	-	-	36	35	-	35	32	40	30	34
Avg Pain	0	2	0	2	0	-	0	0	1	0	1
EMG		No Δ	No Δ	No Δ	L triceps/ biceps	-	L triceps/ biceps	L triceps/ biceps, R FDI	L triceps/ biceps, R FDI	L triceps/ biceps, R FDI	L triceps/ biceps, R FDI
BMCA		No Δ	No Δ	L/R wrist ext	R wrist ext	-	R wrist ext	R wrist ext	R wrist ext	R wrist ext	R wrist ext
