# Supplementary material for: Long-term safety and clinical outcomes from a single-site phase 1 study of neural stem cell transplantation for chronic cervical spinal cord injury
Source: Stem Cell Reports. 2026 May 21;21(6):102926. doi: 10.1016/j.stemcr.2026.102926 (PMC13261865; doi:10.1016/j.stemcr.2026.102926)
Supplement: Document S2. Article plus supplemental information [file mmc2.pdf]

STEM CELL REPORTS
Article
ISSCR
OPEN ACCESS

Long-term safety and clinical outcomes from a single-site phase 1 study of neural stem cell transplantation for chronic cervical spinal cord injury

Mickey E. Abraham,[1] Joel R. Martin,[2] Margaret Seaton,[1] Michael G. Brandel,[1] Kiefer J. Forseth,[1] Catriona Jamieson,[3] Martin Marsala,[4] and Joseph D. Ciacci[1,5,*]
[1]Department of Neurological Surgery, University of California, San Diego, La Jolla, CA 92037, USA
[2]Department of Neurological Surgery, Orlando Health, Orlando, FL 32806, USA
[3]Department of Medicine, Division of Regenerative Medicine and CIRM Alpha Stem Cell Clinic, University of California, San Diego, La Jolla, CA 92037, USA
[4]Department of Anesthesiology, University of California, San Diego, La Jolla, CA 92037, USA
[5]Lead contact
*Correspondence: jciacci@ucsd.edu
https://doi.org/10.1016/j.stemcr.2026.102926

SUMMARY

Neural stem cell transplantation offers a promising avenue for spinal cord injury (SCI) repair, but demonstrating safety and feasibility in human populations remains a critical step. In this phase 1 trial, we investigate the long-term outcomes of cervical spinal cord implantation of NSI-566, a human spinal cord-derived neural stem cell line. With prospective data collection over 60 months, we assess adverse events, functional outcomes, and neurophysiological measures in a small cohort ($N = 3$) of chronic AIS-A SCI patients. We report a favorable safety profile with no evidence of tumorigenicity or procedure-related complications. Subclinical neurophysiological changes and limited functional gains were observed, although interpretation is constrained by the absence of a control group and the confounding effects of post-operative rehabilitation. These findings support the feasibility of intraparenchymal stem cell delivery for chronic SCI and underscore the need for larger, controlled trials to clarify efficacy and optimize therapeutic parameters.

INTRODUCTION

Traumatic spinal cord injury (SCI) is a severe medical condition, with ~18,000 new cases reported each year in the United States (National Spinal Cord Injury Statistical Center, 2025; Cadotte and Fehlings, 2011; Courtine and Sofroniew, 2019; Hachem et al., 2017; Karsy et al., 2019). Cervical SCI can lead to particularly challenging functional deficits, including upper extremity weakness and sensory loss based on neurological level of injury as well as various degrees of autonomic, cardiovascular, and respiratory disruption (Krassioukov, 2009; Javeed et al., 2024; RG et al., 2022; Walters et al., 2013).

NSI-566, a proprietary human spinal cord-derived neural stem cell line, is an allogeneic cell therapy candidate authorized by the US Food and Drug Administration (FDA) for clinical trials under an Investigational New Drug application (Guo et al., 2010). The cell line is derived from a single human fetus and comprises multipotent neural stem cells capable of differentiating into neurons, astrocytes, and oligodendrocytes (Yan et al., 2007). NSI-566 was developed to support spinal cord repair following intraparenchymal transplantation by integrating into the host tissue, promoting synaptic connectivity, and enhancing the regeneration of damaged neuronal circuits and has been tested in clinical trials for thoracic SCI, ischemic stroke, and amyotrophic lateral sclerosis (Cizkova et al., 2007; Curtis et al., 2018; Feldman et al., 2014; Glass et al., 2012, 2016; Goutman et al., 2018; Martin et al., 2024; Riley et al., 2012, 2014; Zhang et al., 2019).

It is important to contextualize NSI-566 with other intraparenchymal stem cell therapies for SCI, such as HuCNS-Sc and OPC1. HuCNS-SC, a fetal brain-derived neural stem cell line, was tested in phase 1/2 trials for chronic and subacute cervical and thoracic SCI, demonstrating safety but limited efficacy, leading to early termination of a phase 2 trial due to lack of functional improvement (Curt et al., 2020; Levi et al., 2018, 2019). OPC1, an oligodendrocyte progenitor cell therapy derived from human embryonic stem cells, was developed to promote remyelination and axonal support post-SCI and demonstrated a favorable safety profile and preliminary motor improvements in subacute cervical and acute thoracic SCI with follow-up trials open for enrollment (McKenna et al., 2022; RG et al., 2022).

NSI-566 can differentiate into neurons, astrocytes, and oligodendrocytes, with preclinical studies demonstrating its potential for structural integration, synaptic connectivity, and support of functional improvement in models of chronic SCI, thus offering a novel cellular profile and mechanism of action (Casarosa et al., 2014; Guo et al., 2010). In rodent models of SCI, NSI-566 supported axonal growth and led to functional improvements at 12 weeks versus control (Cizkova et al., 2007; Curtis et al., 2018; Yan et al., 2007). In a first-in-human phase 1 clinical trial for chronic thoracic complete SCI (ClinicalTrials.gov, NCT01772810), NSI-566 transplantation was associated with long-term safety as well as neurophysiological evidence suggestive of subtle motor and sensory improvements up to 60 months post-implantation (Curtis et al., 2018; Martin et al., 2024).

Published by Elsevier Inc. on behalf of International Society for Stem Cell Research.
This is an open access article under the CC BY license (http://creativecommons.org/licenses/by/4.0/).

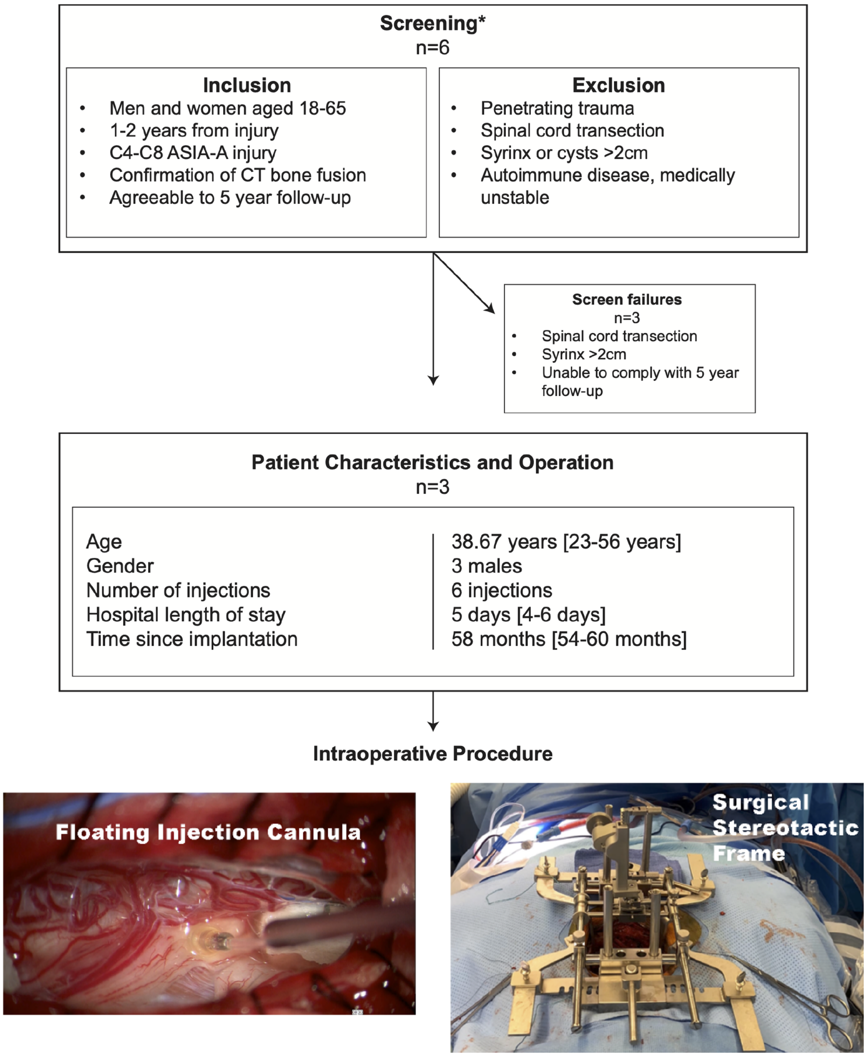

Figure 1. Eligibility screening, patient selection, intra-operative procedure, and perioperative details

The primary objective of this study was to evaluate the safety and feasibility of intraparenchymal implantation of NSI-566 in patients with chronic cervical complete SCI.

RESULTS

Study participants

The study was initiated in 2018 (first dose) and completed enrollment (last dose) in 2019. Of the six individuals screened, three failed to qualify due to exclusion factors including spinal cord transection on MRI, presence of a syrinx >2 cm on MRI, and unwillingness to comply with follow-up assessments for 5 years (Figure 1). All three enrolled participants were white males; one injury was the result of a vehicular accident, one was the result of a fall, and one was the result of a diving accident. The mean age was 38.7 years (23–56 years), and the mean follow-up period was 58 months (54–60 months).

All patients underwent successful surgery without intra-operative complications and completed their post-operative hospital courses uneventfully, with stable neurological status and no procedure-related adverse events (AEs) noted prior to discharge. Upon discharge, all patients resumed outpatient physical therapy, including two to three sessions per week of mobility exercises for 6–12 weeks post-surgery.

Evaluation of safety

Primary endpoint

The primary endpoint, safety, assessed by the incidence of AEs and clinically significant laboratory abnormalities up to 6 months post-transplantation, was successfully

Table 1. Selected outcomes of included subjects 0202, 0203, and 0206

	ISNCSCI	EMG	BMCA
Subject 0202<br>23-year-old male with C7 neurological level of injury sustained in a motor vehicle accident 19 months prior to study enrollment, underwent surgery on March 28, 2018	Overall: no change in overall ISNCSCI at 60 months post-stem cell implantation<br>Motor: one-level improvement from C7 to C8 bilaterally at 42 months through the end of study follow-up<br>Sensory: one-level decrease from C8 to C7 bilaterally at 36 months through the end of study follow-up	No new spontaneous EMG was recorded in the muscle groups below the level of injury	New muscle activation during left wrist extension and flexion tasks at 36 months compared to baseline testing that was sustained through the completion of the study
Subject 0203<br>56-year-old male with C6 neurological level of injury sustained from a fall 23 months prior to study enrollment, underwent surgery on December 12, 2018	Overall: one-level decrease in ISNCSCI from C6 to C5 at 54 months<br>Motor: one-level decrease on the right from C7 to C6, one-level decrease on the left C6–C5 at 54 months<br>Sensory: one-level decrease on the right from C6 to C5 at 54 months, no change on left side	Newly detected MUAPs in the right deltoid at 12 months following cell grafting, with an increase in maximal firing rate from 4.6 to 27 Hz and mean MUAP area from 0.38 to 3.68 μV·ms ($p < 0.001$)	Newly demonstrated improvement in right wrist extension motor activation in the right biceps, triceps, and wrist extension at 12 months compared to baseline (pre-transplant) testing
Subject 0206<br>37-year-old male with C6 neurological level of injury sustained in a diving accident 23 months prior to study enrollment, underwent surgery on March 13, 2019	Overall: no change in overall ISNCSCI at 60 months post-stem cell implantation<br>Motor: no change in motor score at 60 months post-stem cell implantation<br>Sensory: one-level improvement on the right from C6 to C7 at 54 months through the end of study follow-up	Increased-amplitude MUAPs in the right first dorsal interosseous muscle at 42 months post-cell implantation as well as an increase in mean MUAP area in left triceps and biceps recordings at 12 and 36 months, sustained up to 60 months	New well-localized motor responsiveness in wrist extensors bilaterally during voluntary wrist extension tasks compared to baseline testing. On the left, this change occurred at 6 months and subsequently returned to baseline. On the right, increased responsiveness was present from 6 months through the final recording at 30 months

achieved, with all three patients demonstrating no complications during this period (Figure 2).

Secondary endpoints

The results of the secondary endpoints are summarized in Table 1 (International Standards for the Neurological Classification of Spinal Cord Injury [ISNCSCI], electromyography [EMG], and Brain Motor Control Assessment [BMCA]), Figure 3 (ISNCSCI and Spinal Cord Independence Measure [SCIM]), Figure 4 (BMCA), Figure 5 (EMG), and Table S1 (timeline). Follow-up MRI performed serially after transplantation demonstrated no detectable post-cell injection inflammatory changes, swelling, or fluid accumulation suggestive of syrinx formation or any radiographic evidence of tumorigenicity, enlarging mass, spinal cord damage related to the injection procedure, or inflammatory lesions within the spinal cord.

Summary of adverse events

There were no serious AEs related to the surgical intervention or immunosuppressive drugs (Table 2). Each patient experienced various AEs ranging from mild to major, including gastrointestinal complaints ($n = 4$), urinary tract infection ($n = 3$), pain requiring 2–3 days of additional hospitalization ($n = 2$), neurological issues (seizures and dysreflexia), and death. Each AE underwent a thorough root cause analysis to determine its origin and whether it was related to the trial procedures. The most serious AE encountered during the duration of the trial was death. Subject 0203 passed away between the 54- and 60-month postoperative visits, at the age of 60 years, due to cardiac arrest during a vigorous workout. The patient had a documented “do not resuscitate” (DNR) order, which precluded any resuscitative efforts at the time of the incident. Furthermore, the family, in accordance with the subject’s prior wishes, declined an autopsy.

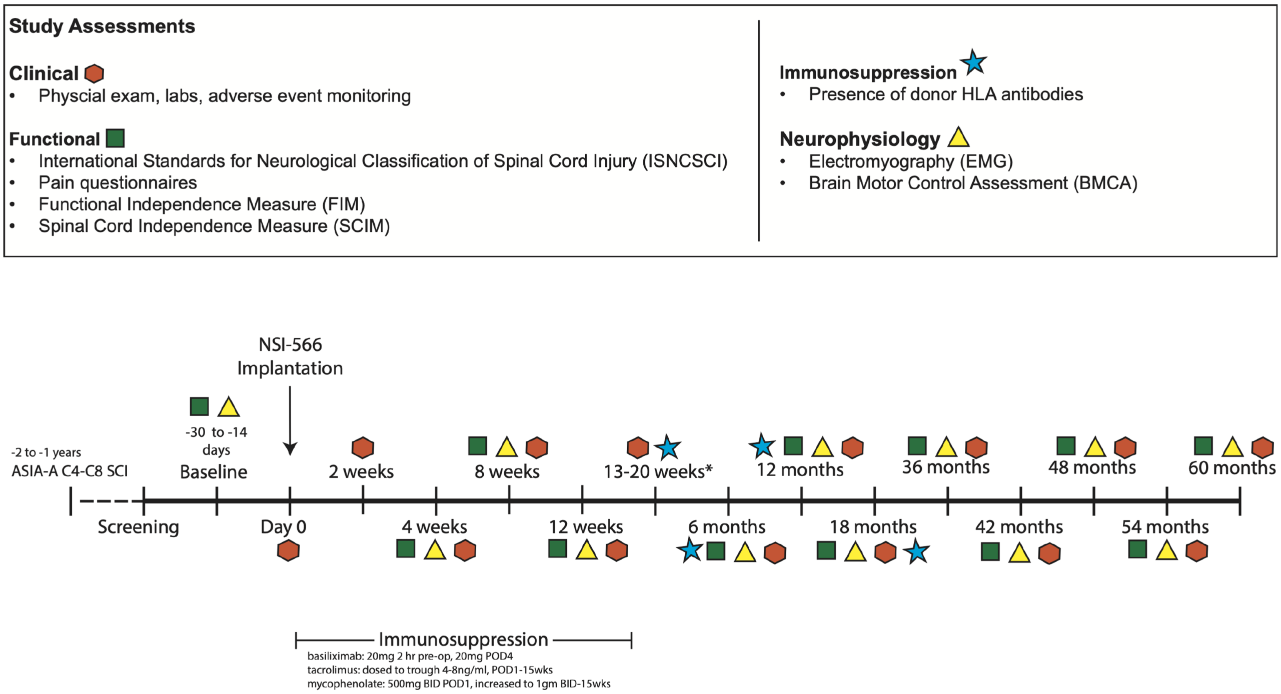

Figure 2. Timeline of study events

*Between 13 and 20 weeks, patients had weekly appointments to monitor immunosuppression response, presence of HLA antibodies, clinical examination, and routine laboratory test results.

Analysis of AE

AE included transient pain in two patients and single episodes of dysreflexia and seizure, all occurring within the first 2 weeks post-implantation. The seizure was self-limited, non-recurring, and occurred during the immediate post-operative period; it was considered possibly related to perioperative stress or post-operative anesthesia regimen, including high-dose fentanyl. The episode of dysreflexia resolved without intervention and was attributed to underlying spinal cord pathology. None of these AEs were attributed to the cell product or immunosuppressive regimen, given their rapid resolution and lack of recurrent or progressive symptoms.

The most serious event during the trial was the death of subject 0203 between the 54- and 60-month follow-up visits, at the age of 60 years and was attributed to cardiac complications during a vigorous workout. While the absence of autopsy limits definitive conclusions, resuscitation was precluded by a documented DNR order, and independent medical review found no evidence implicating the surgical procedure, stem cell product, or immunosuppressive therapy. The presumed cause of death was sudden cardiac arrest during physical exertion, consistent with cardiovascular complications known to be more prevalent in individuals with chronic SCI. This unfortunate death at age 60 years, in the context of the patient's underlying condition and the known reduction in life expectancy associated with SCI, is consistent with a favorable safety profile of NSI-566 transplantation in this limited cohort (Middleton et al., 2012).

DISCUSSION

Five-year safety data have been previously published for the first-in-human trial of direct injection of NSI-566 in patients with chronic thoracic SCI. The current cervical trial was authorized by the FDA following review of the 18-month safety data from the thoracic trial. Targeting the higher cervical injuries may increase both risk and potential benefit, with gains of two or more motor levels offering significant improvements in functional independence (Whiteneck et al., 1999). Notably, all primary and secondary endpoints in this study were prospectively defined and collected per the original clinical trial protocol, enhancing internal validity and enabling a structured, rigorous interpretation of long-term safety and clinical outcomes in this early-phase trial.

The primary safety endpoint at 6 months was met, with AEs consistent with sequela of SCI and post-operative care. While this primary safety endpoint was straightforward, interpreting secondary outcomes, especially aligning ISNCSCI, EMG, and BMCA findings to assess efficacy, along

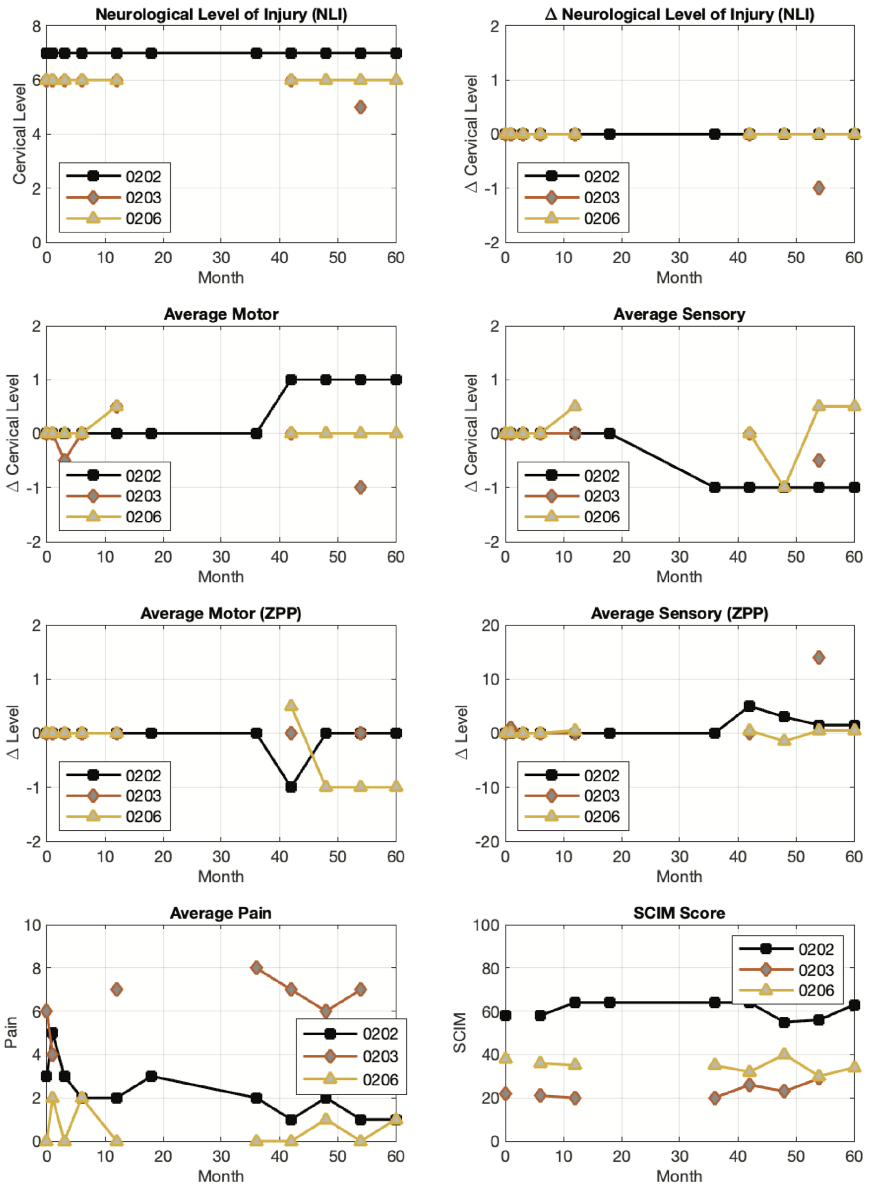

Figure 3. ISNCSCI, average pain, and SCIM results

Neurological level of injury, motor scores, sensory scores, motor and sensory ZPP scores, and pain and SCIM scores for subjects 0206. ZPP, zone of partial preservation; SCIM, Spinal Cord Independent Measure.

with evaluating 60-month safety, was more complex. In subject 0202, a subclinical BMCA improvement at 36 months was not accompanied by corresponding EMG or ISNCSCI changes at that time but was later followed by a positive ISNCSCI motor score change and clinical findings at 42 months. This pattern may reflect the greater sensitivity of BMCA in detecting early or diffuse motor activation that precedes and potentially predicts measurable functional recovery. However, for subject 0203, at 54 months, ISNCSCI motor and sensory scores showed a one-level decline, while BMCA and EMG revealed new or strengthened activity in upper extremity muscles, including wrist extension and deltoid mean unit action potentials (MUAPs), findings inconsistent with the observed clinical decline. Further, subject 0206 showed stable ISNCSCI scores throughout the study, despite EMG and BMCA evidence of subclinical motor improvements, including increased MUAP activity and sustained wrist extension gains. The data of subjects 0203 and 0206 underscore the limited interpretability of neurophysiological improvements in the absence of corresponding clinical change, highlighting the need for caution when considering such findings as evidence of functional recovery and efficacy.

Overall, BMCA scores demonstrated quantifiable improvement for all three patients compared to baseline

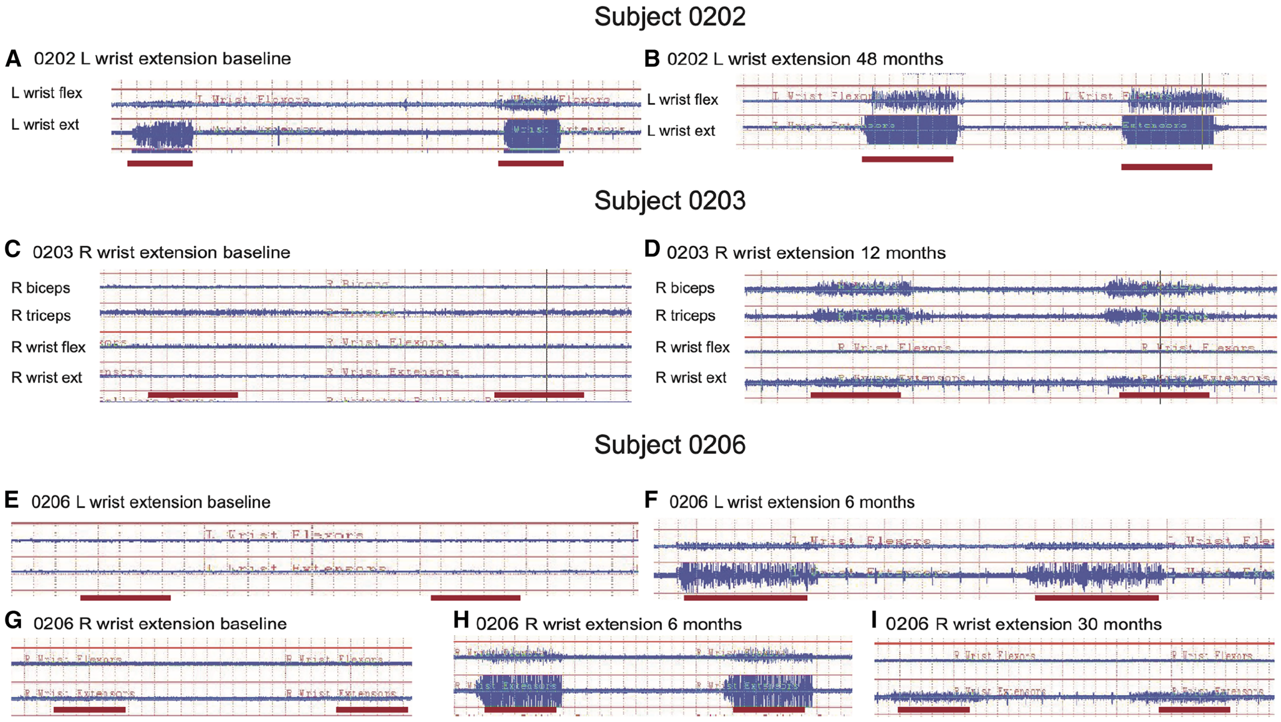

Figure 4. Brain Motor Control Assessment

(A) Upper extremity recording during left wrist extension at baseline.
(B) Upper extremity recording during left wrist extension at 48 months following implantation.
(C) Upper extremity recording during right wrist extension at baseline.
(D) Upper extremity recording during right wrist extension at 12 months following implantation.
(E) Recording of upper extremities in subject 0206 during voluntary left wrist extension at baseline.
(F) Subsequent recording performed at 6 months follow-up.
(G) Upper extremity recording during right wrist extension at baseline.
(H) Upper extremity recording during right wrist extension at 6 months following implantation.
(I) Upper extremity recording during right wrist extension at 30 months.
Red horizontal bars indicate event markers.

(pre-treatment). Prior work comparing BMCA data of neurologically intact patients to those of SCI patients showed that neurologically intact patients can activate the prime movers in each task without involuntary activity in task-irrelevant muscles bilaterally, while SCI patients are unable to localize and, therefore, demonstrate diffuse, uncoordinated activation to discrete tasks (Zoghi et al., 2016). The improved localization in all three subjects suggests a shift toward activation patterns seen in intact patients suggestive of the potential for subclinical recovery.

Notably, all patients underwent two to three sessions per week of mobility exercises for 6–12 weeks post-surgery, which represents a substantial confounder. The data on physical therapy after SCI remain inconsistent with some studies showing functional improvement and others showing no benefit or even harm (Lewis et al., 2022). While functional improvements observed in this study cannot be attributed solely to the transplanted cells and may reflect the effects of post-operative physical therapy, despite participants having reached a recovery plateau prior to enrollment, it is also plausible that the NSC transplantation created a microenvironment conducive to rehabilitation-induced gains, a hypothesis that warrants further investigation. Further, to minimize confounding by spontaneous recovery, all participants were enrolled more than 1 year post-injury, a time frame during which functional gains in patients with complete SCI are exceptionally rare, with less than 2.1% converting from motor complete to motor incomplete status over the subsequent 4 years (Kirshblum et al., 2004).

A comparison of cervical and thoracic NSI-566 implantation outcomes suggests both similarities and important distinctions. In the thoracic cohort, all four participants demonstrated no product-related AEs over 5 years and subclinical neurophysiological improvements on EMG, yet no ISNCSCI motor score gains. In contrast, the current cervical

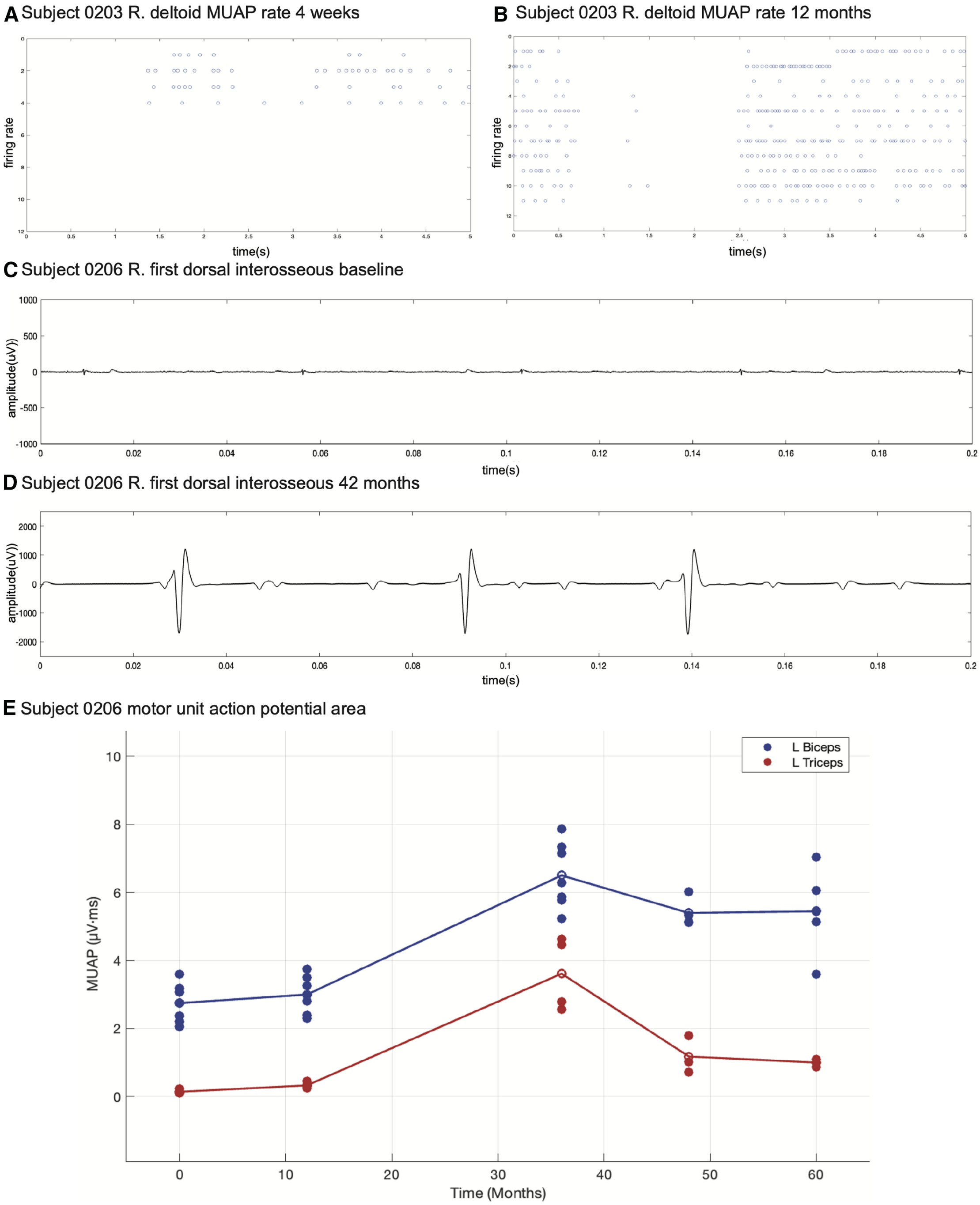

Figure 5. Electromyography following stem cell implantation

(A) MUAP rates measured at 4-week visit from the right deltoid muscle in subject 0203 with a maximum firing rate of 4.6 Hz.

(B) MUAP rates at 12-months follow-up in the right deltoid muscle in subject 0203 showing MUAP rates, with a maximum firing rate of 27 Hz.

(C and D) (C) EMG recording in the right dorsal interosseous prior to stem cell implantation with (D) subsequent right first dorsal interosseous EMG recording at 42 months.

(E) Mean MUAP area (μV*ms) calculated from 5-s epochs at each time point at baseline, 6 months (R deltoid), and 12 months (L triceps, L biceps). Each displayed follow-up shows significant MUAP area increases from baseline. All plots obtained using MATLAB. EMG visualized with 100-Hz high-pass filter. L, left, R, right.

Table 2. All adverse events experienced by subjects 0202, 0203, and 0206

Adverse event	Severity	RTS	RTD	Treatment	Resolution
**0202**					
Diarrhea	Moderate	4	3	Mycophenolate	Resolved, no sequelae
Nausea	Mild	5	4	None	Resolved, no sequelae
Constipation	Mild	5	5	None	Resolved, no sequelae
Coccyx abrasion	Mild	5	5	None	Resolved, no sequelae
UTI	Mild	5	5	None	Resolved, no sequelae
Seizure	Moderate	5	5	None	Resolved with sequelae
**0203**					
UTI	Mild	3	3	Tacrolimus/mycophenolate	Resolved, no sequelae
Pain[a]	Mid	1	5	None	Resolved, no sequelae
Diarrhea	Mild	5	5	None	Resolved, no sequelae
Dysreflexia	Mild	5	5	None	Resolved, no sequelae
UTI	Mild	4	5	None	Resolved, no sequelae
Infected cysts	Mild	5	5	None	Resolved, no sequelae
Death[b]	Severe	5	5	None	Unresolved
**0206**					
Diarrhea	Mild	5	3	Tacrolimus	Resolved, no sequelae
Pain[a]	Mild	1	5	None	Resolved, no sequelae
Pneumonia	Moderate	5	5	None	Resolved, no sequelae

1, definitely related; 2, probably related; 3, possibly related; 4, unlikely related; 5, not related.
UTI, urinary tract infection; RTS, related to study; RTD, related to drug.
[a]Required prolonged pain medication, stayed an additional 2–3 days to manage pain medications and transition to oral medications prior to discharge.
[b]Subject 0203 passed away between the 54- and 60-month post-operative visits due to cardiac arrest during a vigorous workout. The patient had a documented "do not resuscitate" (DNR) order, which precluded any resuscitative efforts at the time of the incident.

cohort exhibited subclinical improvements on EMG and BMCA, but there was also one case (subject 0202) with measurable motor improvement (C7–C8 bilaterally) on ISNCSCI at 42 months. Cervical injuries often retain greater tissue continuity and exhibit more spared axonal pathways, potentially providing a more favorable environment for cell engraftment and synaptic integration, whereas thoracic injuries frequently involve more extensive cavitation and mechanical disruption (Ulndreaj et al., 2017). However, discrepancies across modalities and the small sample size in both studies highlight the need for cautious interpretation and reinforce the importance of larger, controlled trials to clarify efficacy and durability of response across spinal levels.

The most significant limitation of this study is the small sample size ($N = 3$) that substantially limits the generalizability of the findings and precludes robust statistical analysis. In addition, the absence of a control group further restricts the strength of conclusions that can be drawn about efficacy. Another limitation is the unknown optimal timing of stem cell implantation; prior studies have variably targeted the acute, subacute, or chronic phases of SCI, but there is no consensus on a superior time point. Notably, chronic SCI, defined as more than 1-year post-injury, offers the most stable baseline for evaluating interventions because most motor or sensory improvements usually will occur within the year after injury (Kirshblum et al., 2004; McDonald and Sadowsky, 2002). However, there are case reports of improvements after 2 years (Piepmeier and Jenkins, 1988). Thus, it is plausible that the observed minor changes in this cohort may be from spontaneous recovery or as a result of post-surgical physical therapy, with or without a contribution from stem cell transplantation. Additionally, diffusion tensor imaging (DTI) sequences,

which can provide evidence of tract remodeling or improvement, were not utilized in this cervical cohort. This decision was based on significant imaging artifacts caused by spinal instrumentation, as well as inconclusive DTI data from the prior thoracic studies. Furthermore, while combining electrophysiologic data with subjective measures is a relative strength, ISNCSCI scores are susceptible to interrater variability, which may affect data reliability (Armstrong et al., 2017 Nov; Schuld et al., 2015; Snider et al., 2023 Feb 15; Osunronbi and Sharma, 2019). Overall, the observed motor/sensory and neurophysiological changes are exploratory and not indicative of efficacy due to the descriptive nature of the data. An additional consideration is that NSI-566 is derived from a single human fetal tissue source, which raises ethical and societal considerations that, while addressed through regulatory compliance and oversight, may influence the broader acceptance and scalability of this therapeutic approach (Iltis et al., 2023; Lo and Parham, 2009).

This phase 1 trial suggests that intraparenchymal implantation of NSI-566 in cervical SCI is safe and feasible. Future studies can focus on dose escalation in patients with chronic SCI, providing a blinded comparison group, and increasing the number of enrolled patients to enable statistical comparisons. Given the favorable safety profile, it is reasonable to explore transplantation in less-severe ASIA grades, where preserved tissue architecture may facilitate trophic signaling and synaptic relay formation, potentially enhancing therapeutic benefit.

RESOURCE AVAILABILITY

Lead contact

Requests for further information and resources should be directed to and will be fulfilled by the lead contact, Joseph D. Ciacci (jciacci@ucsd.edu).

Materials availability

No new materials were generated in this study.

Data and code availability

No new custom code was generated in this study; however, the scripts used for data analysis are available from the corresponding author upon reasonable request.

ACKNOWLEDGMENTS

This work was supported by Sanford Stem Cell Clinical Center and the CIRM UC San Diego Alpha Stem Cell Clinic.

AUTHOR CONTRIBUTIONS

From the Department of Neurosurgery, UCSD (M.E.A., J.R.M., M.S., M.G.B, K.J.F., and J.D.C.), the CIRM Alpha Stem Cell Clinic, UCSD (C.J.), and the Department of Anesthesiology (M.M.)—all in the United States. M.E.A and J.R.M particpated in the clincal elements of this trial and datal acquisition. M.S. assisted with data analsysis and manuscript preperation. M.G.B. particpated in clinical trial and data collection. K.J.F. particpated in data analysis and manuscript review. C.J. and M.M. particpated in clincal trial design and overview and manuscrupt review. J.D.C. particpated in all all critical roles including funding, clincal trial, and manuscript review.

DECLARATION OF INTERESTS

The authors declare no competing interests.

STAR★METHODS

Detailed methods are provided in the online version of this paper and include the following:

- KEY RESOURCES TABLE
- EXPERIMENTAL MODEL AND STUDY PARTICIPANT DETAILS
  - Clinical trial design and patient selection
  - Eligibility
  - NSI-566 neural stem cell line
  - Outcome measures
  - Study oversight
- METHOD DETAILS
  - Surgical and neural stem cell implantation procedure
- QUANTIFICATION AND STATISTICAL ANALYSIS
- ADDITIONAL RESOURCES

SUPPLEMENTAL INFORMATION

Supplemental information can be found online at https://doi.org/10.1016/j.stemcr.2026.102926.

Received: September 25, 2025
Revised: April 17, 2026
Accepted: April 19, 2026
Published: May 21, 2026

REFERENCES

Armstrong, A.J., Clark, J.M., Ho, D.T., Payne, C.J., Nolan, S., Goodes, L.M., Harvey, L.A., Marshall, R., Galea, M.P., and Dunlop, S.A. (2017). Achieving assessor accuracy on the International Standards for Neurological Classification of Spinal Cord Injury. Spinal Cord *55*, 994–1001. https://doi.org/10.1038/sc.2017.67.

Cadotte, D.W., and Fehlings, M.G. (2011). Spinal cord injury: a systematic review of current treatment options. Clin. Orthop. Relat. Res. *469*, 732–741. https://doi.org/10.1007/s11999-010-1674-0.

Casarosa, S., Bozzi, Y., and Conti, L. (2014). Neural stem cells: ready for therapeutic applications? Mol. Cell. Ther. *2*, 31. https://doi.org/10.1186/2052-8426-2-31.

Chan, A.W., Tetzlaff, J.M., Altman, D.G., Laupacis, A., Gøtzsche, P.C., Krleža-Jerić, K., Hróbjartsson, A., Mann, H., Dickersin, K., Berlin, J.A., et al. (2013). SPIRIT 2013 statement: defining standard protocol items for clinical trials. Ann. Intern. Med. *158*, 200–207. https://doi.org/10.7326/0003-4819-158-3-201302050-00583.

Cizkova, D., Kakinohana, O., Kucharova, K., Marsala, S., Johe, K., Hazel, T., Hefferan, M.P., and Marsala, M. (2007). Functional recovery in rats with ischemic paraplegia after spinal grafting of human spinal stem cells. Neuroscience *147*, 546–560. https://doi.org/10.1016/j.neuroscience.2007.02.065.

Courtine, G., and Sofroniew, M.V. (2019). Spinal cord repair: advances in biology and technology. Nat. Med. *25*, 898–908. https://doi.org/10.1038/s41591-019-0475-6.

Curt, A., Hsieh, J., Schubert, M., Hupp, M., Friedl, S., Freund, P., Huber, E., Pfyffer, D., Sutter, R., Jutzeler, C., et al. (2020). The Damaged Spinal Cord Is a Suitable Target for Stem Cell Transplantation. Neurorehabil. Neural Repair *34*, 758–768. https://doi.org/10.1177/1545968320935815.

Curtis, E., Martin, J.R., Gabel, B., Sidhu, N., Rzesiewicz, T.K., Mandeville, R., Van Gorp, S., Leerink, M., Tadokoro, T., Marsala, S., et al. (2018). A First-in-Human, Phase I Study of Neural Stem Cell Transplantation for Chronic Spinal Cord Injury. Cell Stem Cell *22*, 941–950.e6. https://doi.org/10.1016/j.stem.2018.05.014.

Feldman, E.L., Boulis, N.M., Hur, J., Johe, K., Rutkove, S.B., Federici, T., Polak, M., Bordeau, J., Sakowski, S.A., and Glass, J.D. (2014). Intraspinal neural stem cell transplantation in amyotrophic lateral sclerosis: phase 1 trial outcomes. Ann. Neurol. *75*, 363–373. https://doi.org/10.1002/ana.24113.

Glass, J.D., Boulis, N.M., Johe, K., Rutkove, S.B., Federici, T., Polak, M., Kelly, C., and Feldman, E.L. (2012). Lumbar intraspinal injection of neural stem cells in patients with amyotrophic lateral sclerosis: results of a phase I trial in 12 patients. Stem Cell. *30*, 1144–1151. https://doi.org/10.1002/stem.1079.

Glass, J.D., Hertzberg, V.S., Boulis, N.M., Riley, J., Federici, T., Polak, M., Bordeau, J., Fournier, C., Johe, K., Hazel, T., et al. (2016). Transplantation of spinal cord-derived neural stem cells for ALS: Analysis of phase 1 and 2 trials. Neurology *87*, 392–400. https://doi.org/10.1212/WNL.0000000000002889.

Goutman, S.A., Brown, M.B., Glass, J.D., Boulis, N.M., Johe, K., Hazel, T., Cudkowicz, M., Atassi, N., Borges, L., Patil, P.G., et al. (2018). Long-term Phase 1/2 intraspinal stem cell transplantation outcomes in ALS. Ann. Clin. Transl. Neurol. *5*, 730–740. https://doi.org/10.1002/acn3.567.

Guo, X., Johe, K., Molnar, P., Davis, H., and Hickman, J. (2010). Characterization of a human fetal spinal cord stem cell line, NSI-566RSC, and its induction to functional motoneurons. J. Tissue Eng. Regen. Med. *4*, 181–193. https://doi.org/10.1002/term.223.

Hachem, L.D., Ahuja, C.S., and Fehlings, M.G. (2017). Assessment and management of acute spinal cord injury: From point of injury to rehabilitation. J. Spinal Cord Med. *40*, 665–675. https://doi.org/10.1080/10790268.2017.1329076.

Iltis, A.S., Koster, G., Reeves, E., and Matthews, K.R.W. (2023). Ethical, legal, regulatory, and policy issues concerning embryoids: a systematic review of the literature. Stem Cell Res. Ther. *14*, 209. https://doi.org/10.1186/s13287-023-03448-8.

Javeed, S., Zhang, J.K., Greenberg, J.K., Botterbush, K., Benedict, B., Plog, B., Gupta, V.P., Dibble, C.F., Khalifeh, J.M., Wen, H., et al. (2024). Impact of Upper Limb Motor Recovery on Functional Independence After Traumatic Low Cervical Spinal Cord Injury. J. Neurotrauma *41*, 1211–1222. https://doi.org/10.1089/neu.2023.0140.

Karsy, M., Hawryluk, G., Karsy, M., and Hawryluk, G. (2019). Modern Medical Management of Spinal Cord Injury. Curr. Neurol. Neurosci. Rep. *19*, 9–19. https://doi.org/10.1007/s11910-019-0984-1.

Krassioukov, A. (2009). Autonomic function following cervical spinal cord injury. Respir. Physiol. Neurobiol. *169*, 157–164. https://doi.org/10.1016/j.resp.2009.08.003.

Kirshblum, S., Millis, S., McKinley, W., and Tulsky, D. (2004). Late neurologic recovery after traumatic spinal cord injury. Arch. Phys. Med. Rehabil. *85*, 1811–1817. https://doi.org/10.1016/j.apmr.2004.03.015.

Levi, A.D., Anderson, K.D., Okonkwo, D.O., Park, P., Bryce, T.N., Kurpad, S.N., Aarabi, B., Hsieh, J., and Gant, K. (2019). Clinical Outcomes from a Multi-Center Study of Human Neural Stem Cell Transplantation in Chronic Cervical Spinal Cord Injury. J. Neurotrauma *36*, 891–902. https://doi.org/10.1089/neu.2018.5843.

Levi, A.D., Okonkwo, D.O., Park, P., Jenkins, A.L., Kurpad, S.N., Parr, A.M., Ganju, A., Aarabi, B., Kim, D., Casha, S., et al. (2018). Emerging Safety of Intramedullary Transplantation of Human Neural Stem Cells in Chronic Cervical and Thoracic Spinal Cord Injury. Neurosurgery *82*, 562–575. https://doi.org/10.1093/neuros/nyx250.

Lewis, N.E., Tabarestani, T.Q., Cellini, B.R., Zhang, N., Marrotte, E.J., Wang, H., Laskowitz, D.T., Abd-El-Barr, M.M., and Faw, T.D. (2022). Effect of Acute Physical Interventions on Pathophysiology and Recovery After Spinal Cord Injury: A Comprehensive Review of the Literature. Neurospine *19*, 671–686. https://doi.org/10.14245/ns.2244476.238.

Lo, B., and Parham, L. (2009). Ethical issues in stem cell research. Endocr. Rev. *30*, 204–213. https://doi.org/10.1210/er.2008-0031.

Martin, J.R., Cleary, D., Abraham, M.E., Mendoza, M., Cabrera, B., Jamieson, C., Marsala, M., and Ciacci, J.D. (2024). Long-term clinical and safety outcomes from a single-site phase 1 study of neural stem cell transplantation for chronic thoracic spinal cord injury. Cell Rep. Med. *5*, 101841. https://doi.org/10.1016/j.xcrm.2024.101841.

McDonald, J.W., and Sadowsky, C. (2002). Spinal-cord injury. Lancet *359*, 417–425. https://doi.org/10.1016/S0140-6736(02)07603-1.

McKenna, S.L., Ehsanian, R., Liu, C.Y., Steinberg, G.K., Jones, L., Lebkowski, J.S., Wirth, E., Fessler, R.G., McKenna, S.L., Ehsanian, R., et al. (2022). 04-01). Ten-year safety of pluripotent stem cell transplantation in acute thoracic spinal cord injury. J. Neurosurg. Spine *37*, 321–330. https://doi.org/10.3171/2021.12.SPINE21622.

Middleton, J.W., Dayton, A., Walsh, J., Rutkowski, S.B., Leong, G., and Duong, S. (2012). Life expectancy after spinal cord injury: a 50-year study. Spinal Cord *50*, 803–811. https://doi.org/10.1038/sc.2012.55.

National Spinal Cord Injury Statistical Center. (2025). Facts and Figures at a Glance. https://bpb-us-w2.wpmucdn.com/sites.uab.edu/dist/f/392/files/2025/02/2025-Facts-and-Figures.pdf.

Piepmeier, J.M., and Jenkins, N.R. (1988). Late neurological changes following traumatic spinal cord injury. J. Neurosurg. *69*, 399–402. https://doi.org/10.3171/jns.1988.69.3.0399.

RG, F., R, E., CY, L., GK, S., L, J., JS, L., ED, W., and SL, M. (2022). A phase 1/2a dose-escalation study of oligodendrocyte progenitor cells in individuals with subacute cervical spinal cord injury. J. Neurosurg. Spine *37*, 812–820. https://doi.org/10.3171/2022.5.SPINE22167.

Riley, J., Federici, T., Polak, M., Kelly, C., Glass, J., Raore, B., Taub, J., Kesner, V., Feldman, E.L., and Boulis, N.M. (2012). Intraspinal stem cell transplantation in amyotrophic lateral sclerosis: a phase I safety trial, technical note, and lumbar safety outcomes. Neurosurgery *71*, 405–416. [discussion: 416]. https://doi.org/10.1227/NEU.0b013e31825ca05f.

Riley, J., Glass, J., Feldman, E.L., Polak, M., Bordeau, J., Federici, T., Johe, K., and Boulis, N.M. (2014). Intraspinal stem cell transplantation in amyotrophic lateral sclerosis: a phase I trial, cervical microinjection, and final surgical safety outcomes. Neurosurgery *74*, 77–87. https://doi.org/10.1227/NEU.0000000000000156.

Schuld, C., Franz, S., van Hedel, H.J.A., Moosburger, J., Maier, D., Abel, R., van de Meent, H., Curt, A., Weidner, N., EMSCI study group, et al. (2015). International standards for neurological classification of spinal cord injury: classification skills of clinicians versus computational algorithms. Spinal Cord *53*, 324–353. https://doi.org/10.1038/sc.2014.221.

Snider, B.A., Eren, F., Reeves, R.K., Rupp, R., and Kirshblum, S.C. (2023). The International Standards for Neurological Classification of Spinal Cord Injury: Classification Accuracy and Challenges. Top. Spinal Cord Inj. Rehabil. *29*, 1–15. https://doi.org/10.46292/sci22-00036.

Osunronbi, T., and Sharma, H. (2019). International Standards for Neurological Classification of Spinal Cord Injury: factors influencing the frequency, completion and accuracy of documentation of neurology for patients with traumatic spinal cord injuries. Eur. J. Orthop. Surg. Traumatol. Orthop. Traumatol. *29*, 1639–1648. https://doi.org/10.1007/s00590-019-02502-7.

Ulndreaj, A., Badner, A., and Fehlings, M.G. (2017). Promising neuroprotective strategies for traumatic spinal cord injury with a focus on the differential effects among anatomical levels of injury. F1000Res. *6*, 1907. https://doi.org/10.12688/f1000research.11633.1.

Walters, B.C., Hadley, M.N., Hurlbert, R.J., Aarabi, B., Dhall, S.S., Gelb, D.E., Harrigan, M.R., Rozelle, C.J., Ryken, T.C., Theodore, N., et al. (2013). Guidelines for the Management of Acute Cervical Spine and Spinal Cord Injuries: 2013 Update. Neurosurgery *60*, 82–91. https://doi.org/10.1227/01.neu.0000430319.32247.7f.

Whiteneck, G., Tate, D., and Charlifue, S. (1999). Predicting community reintegration after spinal cord injury from demographic and injury characteristics. Arch. Phys. Med. Rehabil. *80*, 1485–1491. https://doi.org/10.1016/s0003-9993(99)90262-9.

Yan, J., Xu, L., Welsh, A.M., Hatfield, G., Hazel, T., Johe, K., and Koliatsos, V.E. (2007). Extensive neuronal differentiation of human neural stem cell grafts in adult rat spinal cord. PLoS Med. *4*, e39. https://doi.org/10.1371/journal.pmed.0040039.

Zhang, G., Li, Y., Reuss, J.L., Liu, N., Wu, C., Li, J., Xu, S., Wang, F., Hazel, T.G., Cunningham, M., et al. (2019/10/01). Stable Intracerebral Transplantation of Neural Stem Cells for the Treatment of Paralysis Due to Ischemic Stroke. Stem Cells Transl. Med. *8*, 999–1007. https://doi.org/10.1002/sctm.18-0220.

Zoghi, M., Galea, M., and Morgan, D. (2016). Brain motor control assessment of upper limb function in patients with spinal cord injury. J. Spinal Cord Med. *39*, 162–174. https://doi.org/10.1179/2045772314Y.0000000286.

STAR★METHODS

KEY RESOURCES TABLE

REAGENT or RESOURCE	SOURCE	IDENTIFIER
**Experimental models: cell lines**		
Human fetal spinal cord-derived neural precursor line	Neuralstem Inc.	NSI-566

EXPERIMENTAL MODEL AND STUDY PARTICIPANT DETAILS

Clinical trial design and patient selection

This was a Phase I safety study of human spinal cord-derived neural stem cell transplantation for the treatment of chronic cervical SCI. Chronic SCI was defined as at least one year but no more than two years after traumatic SCI. Three subjects with chronic SCI classified as AIS-A, motor and sensory complete SCI, levels C5-7, who met eligibility criteria were enrolled. No control group was included. Inclusion and exclusion criteria are listed in our preliminary report. The trial was registered on ClinicalTrials.gov. IRB approval was granted by the hospital review board.

This study adhered to the Declaration of Helsinki, ICH Good Clinical Practice guidelines, all relevant regulatory standards and was designed and reported in accordance with the SPIRIT 2013 (Standard Protocol Items: Recommendations for Interventional Trials) guidelines to ensure transparency and completeness in trial protocol development.(Chan et al., 2013).

Eligibility

Eligible participants were adults aged 18 to 65 years with traumatic cervical SCI (C5–C7), classified as AIS-A (motor and sensory complete), and between 12- and 24-month post-injury. Inclusion required stable neurological status, anatomical preservation at the injury site confirmed by MRI, and suitability for open surgical access to the spinal cord. Patients were excluded if they had multiple spinal cord lesions, evidence of complete cord transection, significant traumatic brain injury, severe cardiopulmonary comorbidities, prior intradural neurosurgical interventions at the injury level, or detectable somatosensory-evoked potential conduction across the injury site. All procedures were conducted at a single academic center with demonstrated expertise in SCI and stem cell transplantation. Written informed consent was obtained from all participants prior to enrollment or any study procedures. All subjects received spinal cord injections of human spinal cord derived neural stem cells (NSI-566).

NSI-566 neural stem cell line

NSI-566 is a human spinal cord-derived neural stem cell line that was derived from a single postmortem spinal cord of an eight-week gestational age fetus. This tissue was obtained in compliance with the National Institutes of Health (NIH) and Food and Drug Administration (FDA) Good Tissue Practice Guidelines, and under a protocol approved by an outside independent review board. Neural stem cells were isolated by dissociating a single piece of spinal cord tissue of lower cervical/upper thoracic region and expanding it as a single line.

For cell administration, NSI-566 was provided as a live-cell suspension that required no further manipulation. The cell suspension was prepared one day prior to each scheduled surgery at a cGMP facility with a final concentration of $2x10^6$ cells/mL of hibernation medium. This target concentration had been established for being safe and adequate for intraspinal injections by series of preclinical and clinical studies. The cell suspension was then shipped to the surgery site for overnight delivery by a commercial package courier.

Before proceeding with cell administration, the cells suspension was inspected for cell viability to proceed with the implantation. The clinical lot of NSI-566 had undergone extensive preclinical safety and efficacy studies in various small and large animal studies, which had been reviewed by the US FDA under an IND (Investigational New Drug) application (#014413).

Each subject received total of six intraspinal injections (2×10^5 cells/injection delivered in 10 μL of hibernation buffer). The injections were placed bilaterally into the remaining tissue lateral to the injury site and within the medial white matter-appearing tracts of approximately one segment below the injury site, as verified by intra-operative fluoroscopy imaging. Injections were made using a customized stereotactic cell injection device [49].

Outcome measures

Primary outcome measures included adverse events and clinically significant laboratory abnormalities. Additional secondary outcome assessments were made to measure any post-operative changes. Quality of life scores and physical exams were conducted, including ISNCSCI (International Standards for the Neurological Classification of Spinal Cord Injury), SCIM (Spinal Cord Independence Measure), Functional Independence Measure (FIM), allodynia and neuropathic pain, and bowel and bladder follow-up. Neurophysiological changes were monitored when feasible by needle electromyography (EMG) and/or surface poly-electromyography Brain Motor Control Assessment (BMCA). The effectiveness of immunosuppression was determined by absence of donor-specific HLA antibodies. Subjects were followed postoperatively at two weeks, monthly for six months and at every six months for up for total 60 months post stem cell treatment. Patients did not receive any additional rehabilitation beyond their routine outpatient physical and occupational therapy.

Study oversight

An independent Data Safety Monitoring Board (DSMB) was convened at approximately four-week intervals to review the available safety data. The DSMB was tasked with making specific recommendations regarding study continuation. It did not identify any safety issues which precluded continuation of the study.

METHOD DETAILS

Surgical and neural stem cell implantation procedure

The procedure was performed under general anesthesia with patients positioned prone and secured in three-point Mayfield fixation. All participants underwent surgical exposure of the spinal cord, including laminectomy and removal of prior instrumentation, to enable direct access to the injury site. After spinal exposure, a durotomy was performed to visualize the injured segment.

NSI-566 was prepared in a cGMP facility at a final concentration of 2×10^6 cells/mL and shipped overnight for surgical use. NSI-566 was administered via six bilateral intraparenchymal injections using a custom-built stereotactic injection platform anchored to percutaneous posts. A beveled needle attached to a microprocessor-controlled syringe pump to deliver the cells. The injection flow rate was set at 5.0 μL/min, and the needle was maintained *in situ* for one-minute post-injection to minimize reflux. Injections were placed bilaterally into regions of spinal cord parenchyma adjacent to the injury, targeting medial white matter tracts approximately one spinal segment caudal to the injury epicenter. The guide sheath's conversion to a floating cannula enabled consistent delivery depth (~4 mm) and minimized respiratory-related displacement during cell delivery. Injection placement was confirmed intraoperatively using fluoroscopic imaging to ensure accurate rostrocaudal and lateral alignment relative to the lesion margins, allowing for precise and reproducible targeting across participants. Following cell delivery, the dura was closed watertight, and the surgical site was closed in anatomical layers.

All subjects received perioperative prophylactic antibiotics and standard venous thromboembolism prevention measures of subcutaneous heparin (5000U every 8 h). All subjects received a 12-week immunosuppressive regimen beginning with basiliximab (20 mg IV within two hours pre-surgery and again on post-op day 3), followed by tacrolimus (0.1 mg/kg orally every 12 h, maintaining trough levels of 4–8 ng/mL) and mycophenolate mofetil (initially 500 mg twice daily, increased to 1.5 g/day on day 8 and to 2 g/day on day 15), with both agents tapered starting at week 13 and fully discontinued by week 15; donor-specific HLA antibodies were regularly monitored throughout. Antibody surveillance was conducted to monitor for donor-specific HLA sensitization. Safety and clinical outcomes, including International Standards for Neurologic Classification of SCI (ISNCSCI), Spinal Cord Independence Measure (SCIM), Functional Independence Measure (FIM), electromyography (EMG) and Brain Motor Control Assessment (BMCA), were assessed regularly over 60 months.

QUANTIFICATION AND STATISTICAL ANALYSIS

The trial was not powered for formal statistical testing due to the small sample size and absence of a control group. All analyses were descriptive and intended to be exploratory and hypothesis-generating rather than definitive. No

imputation was performed for missing data. Functional assessments, including ISNCSCI scores, SCIM, FIM, EMG, and BMCA, were reported as observed without inferential statistics. The reporting of *p*-values for EMG trends, though occasionally included to highlight intra-subject changes, should be understood in the context of an underpowered, exploratory design.

ADDITIONAL RESOURCES

The trial was registered in ClinicalTrials.gov as NCT01772810 and supported by the UC San Diego Sanford Stem Cell Clinical Center and the CIRM UC San Diego Alpha Stem Cell Clinic.

Stem Cell Reports, Volume *21*

Supplemental Information

Long-term safety and clinical outcomes from a single-site phase 1 study of neural stem cell transplantation for chronic cervical spinal cord injury

Mickey E. Abraham, Joel R. Martin, Margaret Seaton, Michael G. Brandel, Kiefer J. Forseth, Catriona Jamieson, Martin Marsala, and Joseph D. Ciacci

Supplemental Table 1. ***Clinical outcomes of included patients.*** NLI=Neurological Level of Injury; FIM=Functional Independence Measure (out of 128); SCIM=Spinal Cord Independence Measure (out of 100); "-" indicates testing was not done

	Baseline	*4wk*	*12wk*	*6m*	*12m*	*18m*	*36m*	*42m*	*48m*	*54m*	*60m*
0202											
ISNCSCI											
R Sensory	C8	C8	C8	C8	C8	C8	C7	C7	C7	C7	C7
L Sensory	C8	C8	C8	C8	C8	C8	C7	C7	C7	C7	C7
R Motor	C7	C7	C7	C7	C7	C7	C7	C8	C8	C8	C8
L Motor	C7	C7	C7	C7	C7	C7	C7	C8	C8	C8	C8
NLI	C7	C7	C7	C7	C7	C7	C7	C7	C7	C7	C7
HLA	-	-	Neg	Neg	Neg	Neg	-	-	-	-	-
FIM	100	-	-	106	109	112	-	-	-	-	-
SCIM	58	-	-	58	64	64	64	64	55	56	63
Avg Pain	3	5	3	2	2	3	2	1	2	1	1
EMG		No Δ	No Δ	No Δ	No Δ	No Δ	No Δ	No Δ	No Δ	No Δ	No Δ
BMCA		No Δ	No Δ	No Δ	No Δ	No Δ	L wrist flex/ext	L wrist flex/ext	L wrist flex/ext	L wrist flex/ext	L wrist flex/ext
0203											
ISNCSCI											
R Sensory	C6	C6	C6	C6	C6	-	-	C6	-	C5	-
L Sensory	C6	C6	C6	C6	C6	-	-	C6	-	C6	-
R Motor	C7	C7	C6	C7	C7	-	-	C7	-	C6	-
L Motor	C6	C6	C6	C6	C6	-	-	C6	-	C5	-
NLI	C6	C6	C6	C6	C6	-	-	C6	-	C5	-
HLA	-	-	Neg	Neg	Neg	-	-	-	-	-	-
FIM	58	-	-	58	59	-	-	-	-	-	-
SCIM	22	-	-	21	20	-	20	26	23	29	-
Avg Pain	6	4	-	-	7	-	8	7	6	7	-
EMG		No Δ	No Δ	R deltoid MUAPs	R deltoid MUAPs	-	-	R deltoid MUAPs	-	R deltoid MUAPs	-
BMCA		No Δ	No Δ	No Δ	R biceps, triceps, wrist ext	-	-	R biceps, triceps, wrist ext	-	R biceps, triceps, wrist ext	-
0206											

ISNCSCI											
R Sensory	C6	C6	C6	C6	C6	-	-	C6	C5	C7	C7
L Sensory	C6	C6	C6	C6	C7	-	-	C6	C5	C6	C6
R Motor	C6	C6	C6	C6	C6	-	-	C6	C6	C6	C6
L Motor	C6	C6	C6	C6	C7	-	-	C6	C6	C6	C6
NLI	C6	C6	C6	C6	C6			C6	C6	C6	C6
HLA	-	-	Neg	Neg	Neg	-	-	-	-	-	-
FIM	75	-	-	83	86	-	-	-	-	-	-
SCIM	38	-	-	36	35	-	35	32	40	30	34
Avg Pain	0	2	0	2	0	-	0	0	1	0	1
EMG		No Δ	No Δ	No Δ	L triceps/ biceps	-	L triceps/ biceps	L triceps/ biceps, R FDI	L triceps/ biceps, R FDI	L triceps/ biceps, R FDI	L triceps/ biceps, R FDI
BMCA		No Δ	No Δ	L/R wrist ext	R wrist ext	-	R wrist ext	R wrist ext	R wrist ext	R wrist ext	R wrist ext
